# Medical Opinion and Movement

**Published:** 1909-05-22

**Authors:** 


### Medical Opinion and Movement.

Since Blood Counts, and especially leucocyte counts, were advocated a few years ago by many surgeons as an aid in the diagnosis of various forms of suppuration, a great deal of research has been published on the value of leucocyte counts in Appendicitis. As a general rule the conclusions reached have been strongly in favour of trusting to this method of gauging the progress towards resolution or the reverse of inflammatory affections in this viscus; but notwithstanding this the most practical and experienced surgeons, in Britain at least, have fought distinctly shy of the method. The enthusiasm of the clinical pathologist for strictly laboratory methods of diagnosis is well known, and may possibly explain why the apparently clear and definite indications of the leucocytes are so little regarded by clinicians pure and simple. Dr. Pease has recently contributed to the *Annals of Surgery* an elaborate re-investigation of the matter based on a series of 300 consecutive cases, and he comes to conclusions which are worth the attention of those who have to treat appendicitis. All that can be said from leucocyte counts alone is, he believes, that the majority of cases with over 15,000 leucocytes per cm. are severe, and the majority under 15,000 are mild; but there are so many cases of both types that do not conform to the rule that he prefers to rely on the other signs of the case. Similarly he pronounces against Dr. Gibson's rules based on the proportions of the total number of leucocytes to the percentage of polymorphonuclears, and against other systems of interpreting blood counts in appendicitis. It is best, he thinks, not to draw any hard and fast rules from blood counts.

---

The suggestion has recently been advanced that the mysterious condition of Retro-bulbar Optic Neuritis may sometimes be caused by inflammation and suppuration of the accessory sinuses of the nose. At a recent meeting of the Laryngological Section of the Royal Society of Medicine two patients were shown by Dr. Dundas Grant in whom obstinate retro-bulbar neuritis appeared to be beneficially affected by removal of hypertrophied turbinals for the bettering of access to the sphenoidal and posterior ethmoidal cells. Dr. Logan Turner, at a discussion last July at the British Medical Association's Sheffield meeting, was almost disposed to regard these sinuses as accessory to the orbit rather than to the nasal chambers, and pointed out the direct intercommunication of veins, which exists. Professor Onodi, who has investigated the anatomy of the osseous partition between the sinuses and the optic canal, reports actual dehiscence in this septum in more than one per cent. of individuals; and the bony partition is often of extreme thinness, especially when it is formed by the wall of the posterior ethmoidal cell. Dr. Hawthorne, in discussing Dr. Grant's cases, emphasised the difficulty in explaining how a sinus abscess affecting the optic nerve can pick out the maculo-papillary bundle alone. Fuchs would assume a peculiar vulnerability of these fibres, connected with their especially delicate function; but this solution of the problem can scarcely be described as convincing. Dr. Dundas Grant sums up his article on the subject in the *Journal of Laryngology, Rhinology, and Otology* by concluding that in all cases of this affection, not subsiding spontaneously nor due to disseminate sclerosis, the nasal condition should be carefully considered.

---

Mr. H. W. M. Gray describes in the *Medical Magazine* his experience of some of the recent modifications in the Operative Treatment of Varicose Veins which have been invented by American surgeons. The underlying principle of all these procedures, the first of which was introduced by Keller in 1905, is the extraction of the whole length of a varicose vein without the necessity for a long incision. The method which Mr. Gray finds most useful is that of W. W. Babcock, published nearly two years ago. A thick flexible copper wire of twenty-six inches length is used, and it is provided with acorn-like expansions at either end. A short incision is made over the saphenous vein near the saphenous opening, and the vein is lifted out and clamped. Distally to the forceps an incision is then made into the vein, and the smaller of the two bulbs of the extractor is at once slipped in: this prevents hæmorrhage. The instrument is then pushed down the vein carefully, and by a little manipulation can often be made to pass down to the ankle. When the upper bulb is nearly engaged, the vein is ligatured tightly round the copper shaft just below it; another ligature is applied above the pressure-forceps, and the vein is cut below them. The lower bulb is then located and cut down on: the vein is clamped and cut below it, the bulb pushed out and traction made upon it. In this way the whole vein is forcibly pulled out of the lower incision, the tributaries being torn across about an inch from the junctions with it. Hæmorrhage is slight and easily controlled by pressure. The operation is contra-indicated for thrombosed or very thin veins, but otherwise Mr. Gray reports favourably upon it.

---

The Determination of Arterial Blood-Pressure by hæmomanometric instruments has become so common that especial interest attaches to certain observations of Dr. O. K. Williamson lately communicated to the Royal Society of Medicine. It is found that in the case of those with normal or subnormal blood-pressure the readings are practically identical whether the arm or leg be selected for the determination; the average difference is but 2 mm. of mercury. In those, however, with high blood-pressure the (systolic) readings taken from the leg are much higher than those from the arm; the average difference is 32 mm. Furthermore, the higher the blood-pressure the greater is the difference between the limbs in this respect. On the other hand, the diastolic readings reveal no appreciable difference of this nature. The observations were all made with the patient recumbent and with the instrument at the level of the heart. The conclusion arrived at is that the resistance of the arterial wall must be the factor underlying the effects noted, and that this resistance is a direct result of the increased blood-pressure. The legs are in everyday life subjected to a greater hydrostatic pressure than the arms, from the higher column of blood they support, and this is assumed to explain the earlier degeneration of the vessels there. In the subsequent discussion there was evinced a decided disposition to criticise both Dr. Williamson's conclusions and his figures.

PROFESSOR BOAS, of Berlin, has adopted a new method of Treatment for Hæmorrhoids, which is directly opposed in principle to the orthodox ideas on the subject. In cases of prolapsed hæmorrhoids the first step in the treatment, according to classic methods, is to reduce and replace them within the anus. The professor considers this far from rational, and is of opinion that it favours ulceration and hæmorrhage, and leads to the development ultimately of hæmorrhoidal nodules. On the other hand, examination of the anal region will frequently reveal small hæmorrhoidal pockets, which, according to the author, are the remnants of old hæmorrhoids that have become obliterated naturally. Acting upon these ideas, Professor Boas has conceived his new method of treatment, which he terms the extra-anal treatment of hæmorrhoids, and consists briefly in causing a continued prolapse of the hæmorrhoids by which their complete strangulation and obliteration is effected. A preliminary injection of glycerine or sodium chloride solution is made, and the patient is then requested to make sustained bearing down efforts so as to bring the internal hæmorrhoids outside the anal orifice. If necessary the compression cups of Bier may be applied, and prolapse of the hæmorrhoids effected in this way. For the success of the treatment the hæmorrhoids must, of course, remain prolapsed. They then swell considerably and become filled with blood. At the same time the surrounding anal tissue becomes œdematous, and effectually keeps them outside, and also gradually isolates them from the general circulation. After the third or fourth day the hæmorrhoids begin to shrink, and may become slightly ulcerated on the surface, and in the course of the second week they are reduced to small nodules which ultimately disappear. So far the author has only treated eight patients, but has obtained a satisfactory result each time. He states further that the pain and discomfort to the patient during the first few days is not serious, and seldom sufficient even to require morphia or codeine suppositories.

AN interesting report has been made to the Medical Society of Vienna by D. A. von Kutschera, the sanitary inspector-general of Styria, on the Treatment of Cretinism by Thyroid Extract. Since 1907 this treatment has been instituted by the State at the instigation of Professor von Wagner. Thirty-seven stations have been formed at which the cretins are examined. The patients remain at home and are given gratuitously doses of 0.3 gm. daily. The treatment is started as early as possible in life. Idiots and deaf mutes are excluded. Those under three years of age and those who do not tolerate the treatment well are given a half or third of this amount, or even only one dose a week. Altogether 1,011 patients have been treated in this way. Of these 403 have not continued the treatment or have not followed it seriously. According to the author the increase in growth of the child is a good indication, as the other symptoms always improve proportionately. This has been carefully watched in 440 cretins. Of these, in 45 cases, or 10.2 per cent., the growth was inferior to that of a normal individual, and there was little improvement from the treatment. Eighteen patients (4.1 per cent.) showed a normal growth; and in 377 cases (85.7 per cent.) the growth was above that of a healthy child. The most rapid improvement occurred in children between the ages of one and six. As stated, all the other symptoms improved at the same time—the swelling of the tongue, salivation, eczema, sweats, goitre, and the cretin expression, the walk, speech, and intelligence improved, the fontanelles closed, the teeth appeared, the genital organs developed normally, and the general nutrition and temperament benefited.

AT the same meeting of the Medical Society of Vienna Dr. Mautner gave an account of an interesting case of Hæmophilia, in which a severe attack of hæmorrhage, occasioned by the extraction of a tooth, was eventually stopped by the subcutaneous injection of horse-serum. The patient was a child aged four and a half, and had bled for three days. He became so anæmic that a blood count showed only 1,500,000 corpuscles per cubic millimetre. The hæmorrhage did not yield to the application of ordinary astringents. Twenty cubic centimetres of horse serum were injected subcutaneously, and caused an immediate cessation of the bleeding for several hours. A second injection arrested the hæmorrhage completely, and the pulse, which had become very rapid, slowed down. The child developed a polymorphous erythema, and had a slight rise of temperature in consequence of the injections.

DRS. AUGUSTE LUMIERE and GELIBERT, of Lyons, advocate the Treatment of Acute Gout in the joints by puncture and aspiration of the fluid. They based their treatment on the idea that the gouty affection of a joint was due to the excess of uric acid in the system being localised and concentrated in the joint tissues. This, however, they are unable to confirm, as they have never been able to detect on analysis the smallest trace of uric acid in the fluid drawn off from a gouty joint. Nevertheless, they are so convinced of the therapeutic effects of aspiration that they have continued the treatment, and do not hesitate to appraise its value. The immediate results of aspiration are, a rapid cessation of the pain, sudden fall of the temperature, and a definite termination of the attack. The pain is relieved immediately, and the patient can move the joint without any inconvenience, and considers himself cured. The authors claim, moreover, that after aspiration the attacks become much less frequent, and the treatment prevents the formation of deposits in the joints. On account of the rapidity with which a gouty effusion may become absorbed, they recommend puncture as soon as the presence of fluid in the joint can be definitely diagnosed. It appears that the method has only been applied to cases of gout in the knee joint, and the external margin of the patella is recommended for the site of the puncture. Needless to say, the operation must be carried out under the most aseptic conditions. The fluid aspirated resembles that of an ordinary traumatic synovitis, and contains 0.66 to 13 grammes of urea per litre, but not a trace of uric acid or urate. It is highly toxic. Intravenous injections into rabbits show a toxicity of 10 to 12 c.c. per kilogramme. The authors are of opinion that the toxicity is due to the contained albuminoid substances, and they find that the pyretogenic property is not destroyed by heat.

---

A NEW compound of mercury—the Oleo-brassidate—is described by Raoul Dupuy in *La Revue Thérapeutique Medico-Chirurgicale.* The salt resembles clear yellow jelly. While feebly soluble in hot water, in tepid water to which soap has been added it is completely soluble. It consists of the oleate and brassidate of mercury, representing about 30 per cent. of the metal. Its great advantage over other mercurial ointments lies in the ease with which it can be spread; its independence of any excipient; its rapid absorption by the skin; and its extreme cleanliness in use. As a substitute for the ordinary blue ointment this substance is particularly recommended. It can be used both as a germicide for pediculosis pubis and as an inunction for syphilis. It has been extensively used at the Saint Lazare Hospital with very satisfactory results.

---

IN the *Journal des Sciences Médicales de Lille*, Piet argues in favour of Immediate Amputation for Extensive Crushing of Limbs. Some authorities consider this a blind and dangerous proceeding. Piet, however, declares that amputation is absolutely necessary in certain cases in which the hæmorrhage is extensive and uncontrollable, and points out that an operation lasting barely twenty minutes is not likely to be more dangerous than the prolonged and complicated measures, taking in some cases an hour, which are necessary to disinfect a wound thoroughly. Moreover, infection often follows the most elaborate measures of disinfection; whereas in this type of case suppuration is almost always prevented by immediate amputation. The dangers of insomnia, anorexia and rapid physical deterioration, and of septicæmia can only be avoided, according to Piet, by immediate operation. He maintains, therefore, that if at the outset amputation seems to be indicated, this should be done at once and not postponed until shock has disappeared.

---

MANY years ago Pfeiffer described a complication of Influenza, which he termed Glandular Fever. He considered it a special disease, almost a morbid entity. Nowadays it is no longer recognised as a separate disease, and it is, in fact, found also as a complication of other pyrexias. A mild form of this affection is described by Bureau. This occurs for the most part in children. The onset is rapid, the temperature quickly rising to between 102° and 105° F. At the same time all the symptoms of influenza are noticed—pains in the limbs, vomiting, and anorexia. Soon after is seen a swelling of the glands of the neck, with stiffness of the muscles and difficulty in deglutition. After 48 hours or so the fever commonly subsides, but it may continue as long as ten days, and may show intermissions. Some enlargement of the liver and spleen, with hypogastric pain, are often present. The author attributes this pain to enlargement of the mesenteric glands. Albuminuria also may occur. Suppuration never follows the glandular inflammation. A second variety, more chronic in type, is also found, but it is rare, and often occurs unrecognised. Here the abdominal glands are most liable to be affected, while albuminuria and diarrhœa are common, and considerable wasting is often seen. The bacteriology of this disease is as yet obscure. Streptococci, staphylococci, pneumococcus and the bacillus of influenza are usually found in association; but much more work is needed, in this and other directions, to determine the nature of glandular fever.

---

TRILLAT and Legendre have carefully experimented with Formol with a view to discovering the efficacy of this drug in destroying flies. They have been able to show that it is by the ingestion of the drug that the flies are killed. If Formol be mixed with milk, the flies are attracted to the mixture, and die in large numbers. The fluid should be poured out on a large surface, and need be of no great depth. In such rooms as are infested with flies it is best to put a number of large flat vessels containing a mixture of 15 per cent. of commercial Formol, 20 per cent. of milk, and 65 per cent. of water. The dead bodies of the flies are subsequently found lying at some distance from the receptacles, and not, as might be imagined, in the mixture. In stables and dairy-houses the ground can be sprinkled with skim-milk containing 10 per cent. of Formol. Now that so many diseases have been shown, or strongly suspected, to be disseminated by flies, scientific measures for the destruction of these insects should be recorded, especially at the onset of summer.

---

JAVAL and Boyet showed some time ago that the different Body Fluids, blood-serum, ascitic fluid, pleural effusions, etc, when taken from the same patient at the same time, contain approximately identical quantities of urea, irrespective of the figure of the concentration of this substance. Hence they concluded that it is diffused throughout the organism, and helps to maintain an isotonic condition between the various serous surfaces. Some new experiments described by them confirm their former results, and even justify conclusions with respect to the total amount of nitrogen retained in the system, with the exception of nitrogen contained in albuminoid substances. In five cases they were able to show an almost identical content not only of urea, but of all nitrogen which was of non-albuminous origin, in serous fluids removed at the same time from the same subject. This uniformity of distribution, both of non-albuminous nitrogen and of urea greatly facilitates the discovery of any excess of nitrogen in the organism, since it allows of the choice of the liquid in which to study these two forms of nitrogen retention. In the absence of blood-serum, an equally accurate result can be arrived at from the study of any body fluid such as cerebro-spinal, pleural, or ascitic fluid.

